# Xyloglucan, a Plant Polymer with Barrier Protective Properties over the Mucous Membranes: An Overview

**DOI:** 10.3390/ijms19030673

**Published:** 2018-02-27

**Authors:** Núria Piqué, María del Carmen Gómez-Guillén, María Pilar Montero

**Affiliations:** 1 Department of Microbiology and Parasitology, Pharmacy Faculty, Universitat de Barcelona (UB), Diagonal Sud, Facultat de Farmàcia, Edifici A, Av Joan XXIII, 27-31, 08028 Barcelona, Spain; 2 Institut de Recerca en Nutrició i Seguretat Alimentària de la UB (INSA-UB), Universitat de Barcelona, 08921 Barcelona, Spain; 3 Institute of Food Science, Technology and Nutrition (ICTAN-CSIC), 28040 Madrid, Spain; mc.gomez@csic.es (M.d.C.G.-G.); mpmontero@ictan.csic.es (M.P.M.)

**Keywords:** xyloglucan, diarrhoea, gastroenteritis, nasal obstruction, rhinitis, rhinosinusitis, barrier properties, allergy, preventive measures, medical devices

## Abstract

Disruption of the epithelial barrier function has been recently associated with a variety of diseases, mainly at intestinal level, but also affecting the respiratory epithelium and other mucosal barriers. Non-pharmacological approaches such as xyloglucan, with demonstrated protective barrier properties, are proposed as new alternatives for the management of a wide range of diseases, for which mucosal disruption and, particularly, tight junction alterations, is a common characteristic. Xyloglucan, a natural polysaccharide derived from tamarind seeds, possesses a “mucin-like” molecular structure that confers mucoadhesive properties, allowing xyloglucan formulations to act as a barrier capable of reducing bacterial adherence and invasion and to preserve tight junctions and paracellular flux, as observed in different in vitro and in vivo studies. In clinical trials, xyloglucan has been seen to reduce symptoms of gastroenteritis in adults and children, nasal disorders and dry eye syndrome. Similar mucosal protectors containing reticulated proteins have also been useful for the treatment of irritable bowel syndrome and urinary tract infections. The role of xyloglucan in other disorders with mucosal disruption, such as dermatological or other infectious diseases, deserves further research. In conclusion, xyloglucan, endowed with film-forming protective barrier properties, is a safe non-pharmacological alternative for the management of different diseases, such as gastrointestinal and nasal disorders.

## 1. Background

Nowadays, there is increasing interest in recognizing the fundamental role of the mucosa as a protective barrier to prevent a wide variety of diseases, for which mucosal disruption by bacterial virulence factors, allergens, pro-inflammatory compounds or environmental particles have been identified as primary or contributing pathogenic factors [1,2,3,4]. In this regard, recent data suggest that the epithelium with its tight junctions is considered tight under normal conditions but can be abnormally permeable, with alterations in the tight junctions (changes in expression and localization of tight junction proteins) in pathogenic conditions [2,3,5,6]. 

At intestinal level, an increasing number of studies characterizing the intestinal gut microbiota have shown that perturbations of gut microbiota are associated with the development of different diseases, such as colorectal cancer [7,8], Crohn’s disease [9], irritable bowel syndrome (IBS) [10], neurological [11] or renal diseases [12] and, in most cases, a loss of intestinal epithelial barrier function-produced by pro-inflammatory compounds such as the bacterial lipopolysaccharide LPS has been identified [2,10,12]. At the nasal epithelium, results of in vitro studies suggest that epithelial tight junctions may be affected by protease activities of pollen and house dust mite allergens in allergic rhinitis, leading to an abnormally permeable epithelium with alterations in the tight junctions (changes in expression and localization of tight junction proteins) [3,5,13,14].

In this context, there is increasing interest in non-pharmacological approaches that can contribute to preventing or reversing the mucosal disruption produced by this variety of factors. The objective of these strategies is to create a mechanical barrier over the mucosa, with the aim of reducing contact between allergens, irritants, pathogens and their virulence factors and triggering factors and the mucosa [1,3,6,15,16,17,18]. The use of these barrier protective measures, usually marketed as medical devices, is also supported in the current context of high levels of antimicrobial resistance [19,20,21] and the need to avoid chronic pharmacological treatments and their adverse events [18].

In this review, we present the scientific evidence that supports the use of medical devices containing compounds that act as mucosal protectors, such as xyloglucan (a hemicellulose extracted from the seed of the tamarind tree *Tamarindus indica*) to restore the physiological function of the mucosal epithelial cells, by forming a bio-protective film that prevents contact with pathogens and their products, allergens and pro-inflammatory compounds. In this review, we also highlight those issues for which further research is needed to complete the development of xyloglucan medical devices such as mucosal protectors. We have analysed the results published in PubMed regarding the general chemical properties of xyloglucan, published early in the 1990s, and more recent in vitro and animal studies and clinical trials, without excluding any study performed with xyloglucan.

Xyloglucan is a non-ionic, neutral, branched polysaccharide consisting of a cellulose-like backbone that carries xylose and galactosyl-xylose substituents. The configuration of this polysaccharide gives the product a “mucin-like” molecular structure, thus conferring optimal mucoadhesive properties. These mucoadhesive properties allow xyloglucan to act as physical barrier that protects the integrity of mucosal cells against different damaging agents, such as microorganisms, allergens and pro-inflammatory compounds.

We present the results of in vitro studies in models of intestinal and nasal mucosa and conjunctival cells [1,16,17,22] and in vivo models of intestinal inflammation and diarrhoea, demonstrating its barrier properties against bacterial adhesion and invasion and pro-inflammatory compounds, and the results of clinical studies in which the mucosal protectors have demonstrated their protective effect in patients with gastrointestinal disorders (mainly diarrhoea), urinary tract infections, nasal respiratory diseases and dry eye syndrome [15,23,24,25,26,27,28]. Since medical devices containing mucosal protectors are a new strategy for the management of these diseases, we discuss the data published to date and the issues that deserve further research.

## 2. The Mucin Network and Mucosal Cell Integrity

### 2.1. Mucin Network Thickness, Structure and Properties, Nasal and Intestinal Mucus

Mucin networks are viscoelastic fibrillar aggregates formed through the complex self-association of biopolymeric glucoprotein chains that form a lubricious, hydrated protective shield along epithelial regions within the human body [29]. Through the structural, chemical and barrier properties, these functional coatings play a critical role in maintaining human health. When these coatings are lost, a perceivable dryness along the cornea/dry eyes), buccal cavity (xerostomia) or upper respiratory tract can occur, rendering the epithelium highly vulnerable to pathogens (e.g., intestinal inflammation from loss of gastrointestinal mucus [29].

**Figure 1:**
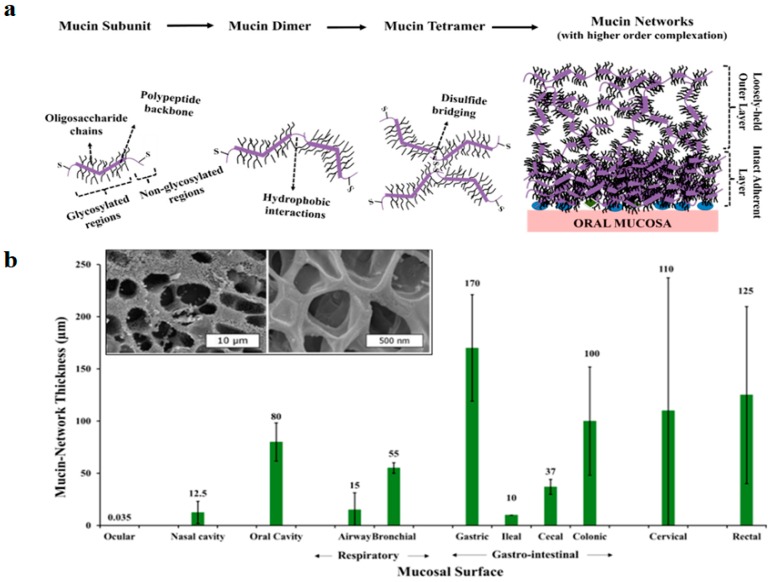
Characteristics of the mucin network from different mucosal surfaces. (**a**) Progression of higher order complexation of mucin glycoproteins resulting in the formation of a mucin network over an oral mucosal surface. This scheme demonstrates the progression of a high-order complexation process, which results in the formation of mucin aggregates. Mucin aggregates invariably contain two-distinct zones: the more intact adherent mucin layers, and loosely-held (expanded) mucin layers of high free-volume. (**b**) Mucin network thickness in the respiratory and gastrointestinal mucosa; thickness is expressed in µm. Mucin network thickness varies according to its physiological location and role. Inset figure shows cryo-SEM imaging of pulmonary mucin, demonstrating heterogeneous mesh size distribution [29].

Mucins are glycoproteins secreted by epithelial goblet cells, and mucus cells present in submucosal glands [29,30]. In general, mucin contains a polypeptide backbone which is predominantly made up of serine, alanine, proline, glycine and threonine and an oligosaccharide side chain decorates the peptide backbone, which is covalently grafted via *O*-glycosidic linkages. Mucin possesses a bottlebrush-like structure. The high molecular weight glycoproteins crosslink via disulphide bonds and form longer branched structures (mucin multimers) (Figure 1A).

Influenced by regional environmental conditions, the structural organization of natural mucin networks can differ regionally and temporally throughout the body [29]. Mucin networks can be visualized as multilayers of mucin glucoproteins. In the respiratory or gastrointestinal tract, the networks contain two distinctive zones: loosely adherent outer layers with an expanded free volume that are prone to easy removal, on which microbiota is mainly present, and a denser, more intact, mucosal adherent inner layer [29].

Mucin network thickness varies according to its physiological location and role, being highly dependent on proximity to the secretory glands and other physical factors, such as breathing, mastication and swallowing. In the human gastrointestinal tract, the mucin network thickness can be highly variable, ranging between 50 and 450 µm, while thinner layers have been described for respiratory mucosal surfaces (Figure 1B).

Contrary to earlier belief, the intestinal epithelial barrier is not a static physical barrier but interacts rather strongly with the gut microbiome and cells of the immune system. This intense communication between epithelial cells, immune cells and microbiome will shape specific immune responses to antigens, balancing tolerance and effector immune functions [31]. For its part, the airway epithelial barrier protects the upper and lower airways against multiple threats, such as infectious agents, allergens and other substances, and the characteristics of this system determine the prognosis of respiratory diseases [32].

### 2.2. Glycans: Importance in Host-Microbial Interactions and Pathogenicity

Results obtained from in vivo mouse models have suggested that the relationships between gastrointestinal bacterial species and their hosts may depend on the availability of specific epithelial cell surface glycans [33]. A remarkable feature of mammalian glycoconjugates is their high structural complexity and diversity. Results of different studies with pathogenic (*Helicobacter pylori*) and commensal bacteria (such as *Bacteroides thetaiotaomicron*) support the hypothesis according which the capacity to synthesize diverse carbohydrate structures may have arisen in part from the need to both prevent pathogenic relationships and to co-evolve symbiotic relationships with the non-pathogenic resident microbes [33].

### 2.3. Role of Tight Junctions on Mucosal Cell Integrity

The mucosal barrier is composed of polarized epithelial cells with distinct apical and basolateral surfaces separated by tight junctions and acts as both a physical and an immunological barrier to incoming pathogens [34]. Tight junctions are the most important component for the construction of a constitutive barrier of epithelial cells, and they regulate the permeability of the barrier by tightly sealing the cell–cell junctions. Tight junction proteins include claudins, occludin, junctional adhesion molecules, and scaffold protein zonula occludens [2]. Among them, claudins are the major components of tight junctions and are responsible for the barrier and the polarity of the epithelial cells [2].

### 2.4. Disruption of Mucosal Barrier Function as a Cause of Diseases

Many human pathogens have virulence mechanisms that target the polarity network to enhance binding, create replication niches, move through the mucosal barrier by transcytosis, or bypass the barrier by disrupting cell–cell junctions [34]. Gastrointestinal diseases, including reflux oesophagitis, inflammatory bowel disease, functional gastrointestinal disorders and cancers, may be regulated by tight junctions, and a disruption of their functions leads to chronic inflammatory conditions and chronic progressive disease [2,6,35,36].

Regarding the airway epithelial barrier, recent data suggest that the epithelium with its tight junctions is considered tight under normal conditions but can be abnormally permeable, with a decreased presence of tight junctions, in pathogenic conditions (the “hyperpermeability hypothesis”), such as allergic rhinitis [3,5,13,14] or rhinosinusitis [37,38], or also in contact with high levels of contamination (such as traffic-related air pollutants) [1,39]. 

## 3. General Properties of Xyloglucan and Other Polymers

### 3.1. Structure

#### 3.1.1. Structure of Xyloglucan

Xyloglucan is a matrix polysaccharide that is present in the cell walls of all land plants [40]. Xyloglucan is a hemicellulose, with side chains containing xylose, galactosyl and fucosyl substituents. Two kinds of xyloglucan have been identified in plants: fucosylated, with a structural role in plant cell walls, and non-fucosylated, such as xyloglucan derived from the tamarind seed, with a storage role [41]. Xyloglucan is widely used as a common additive for food and cosmetics, where it acts as a thickener and a stabilizing agent. Since side chains play an important role in the polymer conformation and, thus, in its interaction with other polysaccharides, different structural-activity relationships have been identified. For example, it has been shown that thiolation of xyloglucan improves its bioadhesion and drug permeation, without affecting the resultant gel properties [42]. Likewise, enzyme-degraded xyloglucan gels have been studied as vehicles for nasal and rectal delivery of drugs [43,44].

**Figure 2:**
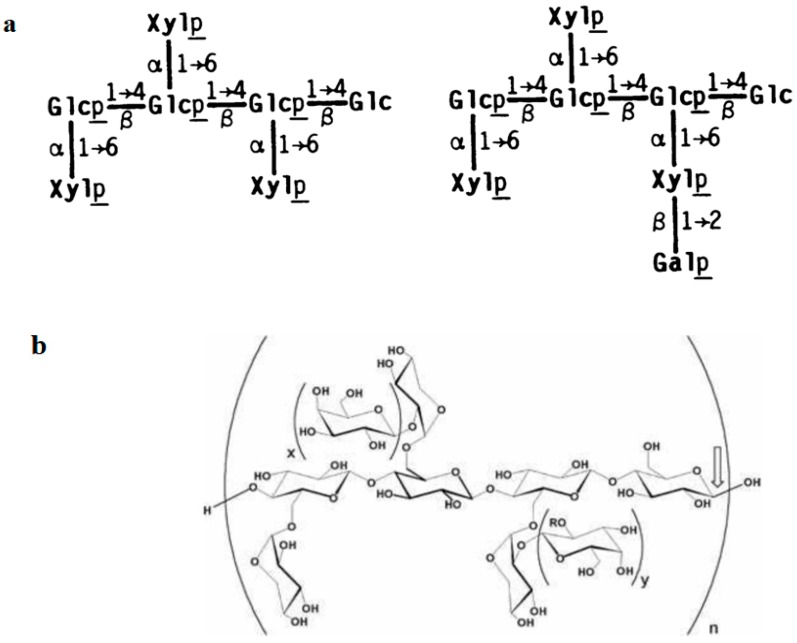
Composition of xyloglucan oligosaccharides extracted from the seed of the tamarind tree (*Tamarindus indica*). (**a**) Structures of xyloglucan. Structures shown are those reported for the quantitatively major oligosaccharides of each size-class isolated from cellulase-digests of xyloglucan [47]. (**b**) Configuration of xyloglucan. The configuration of xyloglucan gives the product a “mucin-like” molecular structure, thus conferring optimal mucoadhesive properties [46].

The xyloglucan used in the medical devices dealt with in this review is a natural biodegradable high-molecular-weight branched polysaccharide (hemicellulose) derived from the tamarind seed. The main component of tamarind seeds has been identified as a non-ionic, neutral, branched polysaccharide consisting of a cellulose-like backbone that carries xylose and galactosyl-xylose substituents (Figure 2). This xyloglucan mainly consists of four types of oligosaccharides as repeating units (heptasaccharide, two varieties of octasaccharide and a nonasaccharide). The monomer unit contains three types of sugars (xylose, galactose and glucose) at a molar ratio of 2.25:1:2.8 [45]. The configuration of this polysaccharide gives the product a “mucin-like” molecular structure, thus conferring optimal mucoadhesive properties [46].

#### 3.1.2. Structure of Synthetic Polymeric Mucin Analogues

Currently, the use of synthetic mucin analogues as a functional model of mucin networks is still in an early exploratory phase [29]. Synthetic networks should be capable of structurally and functionally mimicking bulk properties of natural networks, such as mucoadherence, lubricity and microbial capture/inhibition. As examples, polymeric gels (phenylboronate-salicylhydroxamate-based polymeric systems) have been studied as a synthetic cervicovaginal coating, blocking macrophage migration or inhibiting transport of sexually transmittable pathogens like HIV. For mucus bacterial capture, chitosan and pectin in cross-linked hydrogels have been studied, while custom-synthesized fluorescent glycopolymers containing α- and β-galactose as pendant sugar moieties displayed lectin-mediated binding tendency in human respiratory pathogenic bacteria such as *Pseudomonas aeruginosa* and *Staphylococcus aureus* [29].

#### 3.1.3. Structure of other Mucosal Protectors: Reticulated Proteins

Currently, there is also increasing interest in the use of tannins and reticulated proteins (such as gelatin, etc.) using tannins (forming a three-dimensional complex) due to their film-forming properties [48,49,50].

### 3.2. General Properties of Xyloglucan

#### 3.2.1. Physicochemical Properties

Xyloglucan possesses such properties as high viscosity, in the form of an aqueous solution, broad pH tolerance and adhesiveness. This has led to its application as a stabilizer, solubilizer, thickener, gelling agent and binder in the food and pharmaceutical industries [51,52,53], plus its inclusion in medical devices for the management of gastrointestinal, urinary and nasal disorders due to its film-forming properties that are described in this review.

#### 3.2.2. Solubility

Xyloglucan is highly water-soluble even though its individual chains are not completely hydrated [54], but it is sufficiently water-soluble to form a colloidal dispersion. The presence of xylose single side chains and two ring xylose-galactose combinations has a significant effect on the hydration and solubility of xyloglucan [41].

#### 3.2.3. Assembly

Evaluation of xyloglucan by atomic force microscopy has revealed rod-like nanomolecules of xyloglucan with a mean height of 2.3 ± 0.5 nm and a mean length of 640 ± 360 nm [55]. This study also showed that xyloglucan chains possess a helical structure. The xyloglucan molecules were able to aggregate as cross-like and parallel-like assemblies, and possibly as rope-like structures [55].

#### 3.2.4. Mucoadhesive Polymer

The term bioadhesion alludes to the adherence of molecules to biological membranes, while mucoadhesion refers to adherence specifically to mucin [56]. The configuration of xyloglucan gives the product a “mucin-like” molecular structure, thus conferring optimal mucoadhesive properties [46]. The good mucoadhesive properties of xyloglucan are attributed to the ramifications of xylose and galactoxylose, conferring a configuration similar to mucin [57,58]. The secondary hydroxyl groups present in xyloglucan are principally responsible for mucoadhesion and confer an anionic charge [42].

Xyloglucan possesses high swelling capacity, which is of great importance to initiate the bioadhesion process [59], in its multiple applications, for example as gel-forming ocular films [59]. It has been shown that at the concentrations present in ophthalmic preparations, the structural similarity of xyloglucan to endogenous mucin may allow formulations containing this polymer to adhere readily to the ocular surface for prolonged periods and provide sustained release [46].

#### 3.2.5. Viscosity

Xyloglucan has long been used as a thickener in the food and pharmaceutical industries because its aqueous solution has high viscosity and stability against heat, pH, and shear [60]. Dilute solutions (≤0.5% *w*/*w*) of xyloglucan are characterized by a near Newtonian flow behaviour, whereas higher concentrations (usually higher than 1% *w*/*w*) lead to typical shear-thinning behaviour [61]. With increasing polymer concentration from dilute to semi-dilute conditions, the rise in viscosity is produced when individual polymer coils begin to interpenetrate [62].

By comparing different hemicelluloses, xyloglucan was found to present lower viscosity than galactomannan, resulting from its lower molecular weight and its higher amount of substituted glucose [63]. As a consequence of these molecular properties, the critical concentration of dilute xyloglucan solution is considerably higher than that of galactomannan, i.e., more concentration of xyloglucan would be needed to provide steric and frictional interactions between neighbouring polymer coils.

Xyloglucan is also characterized by presenting viscoelastic properties in solution. The viscoelastic behaviour of xyloglucan dispersions from 0.5% to 1% corresponds to a dilute polymer solution at low oscillation frequencies, in which the viscous modulus (G′′) is larger than the elastic modulus (G′), indicating that the system has an essentially liquid behaviour [61].

## 4. Barrier Properties as Novel Approaches for the Management of Bacterial Infections and Allergy

### 4.1. Anti-Adhesive and Anti-Invasion Properties

Bacterial adherence and subsequent internalization confer advantages to bacteria, facilitating the delivery of virulence factors and toxins, aiding the acquisition of vital nutrients and the evasion of both the host immune system and antibiotics [64]. Anti-adherence strategies are increasingly becoming a source of novel therapeutics to prevent and treat bacterial infectious diseases [64,65]. Anti-adhesion therapies for bacterial infections offer an alternative to antibiotics, in which bacteria are not killed, but are prevented from causing damage to a host by inhibiting adherence to host cells and tissues, a prerequisite for most infectious diseases [64]. Xyloglucan and reticulated proteins are also alternatives with anti-adhesive and anti-invasion properties, as described in this review.

### 4.2. Inhibition of Bacterial Biofilms

Biofilms, typically consisting of densely packed, multispecies populations of cells, encased in a self-synthesized matrix, are the predominant mode of growth for bacteria in most natural, industrial and clinical environments [66]. Recent studies have identified several polysaccharides that inhibit biofilm formation by a wide spectrum of bacteria and fungi, both in vitro and in vivo. None of the anti-biofilm polysaccharides identified to date have been shown to exhibit bacteriostatic or bactericidal activity. Their anti-biofilm activity is therefore likely to be mediated by mechanisms other than growth inhibition [66]. Although the precise mechanism by which polysaccharides can inhibit bacterial biofilms is not completely elucidated [66], polysaccharides with film-forming properties such as xyloglucan could act inhibiting bacterial biofilms by bacterial competition and niche exclusion, although this activity deserves further research.

### 4.3. Preservation of Tight Junctions (Dietary Fibres)

Dysfunction of the mucosal barriers may lead to increased permeability (for example by bacterial LPS or pro-inflammatory compounds) and a pathogen-associated molecular pattern that stimulates innate immune responses in macrophages, neutrophils, endothelial cells, and adipocytes [67]. In this regard, there is increasing interest in the research into products with the capacity to preserve tight junctions, mainly at intestinal level [16,68], but also in other epithelial cells such as the respiratory mucosa [1,69].

At intestinal level, apart from the results obtained with xyloglucan, described in the next section, other dietary fibres have been shown to improve intestinal barrier function through their fermentation products such as short chain fatty acids (SCFAs) [68].

At respiratory level, due to the importance of the airway epithelium as an impermeable barrier (exclusively formed of tight junctions) that protects against inhaled substances and pathogens [37], the research into products like xyloglucan with protective barrier properties is currently acquiring great interest [1,69].

## 5. In Vitro Evidence for the Barrier Properties of Xyloglucan

### 5.1. Effects of Xyloglucan to Preserve Tight Junctions and Paracellular Flux in Caco-Goblet Cells and MucilAir Cells

In a model of intestinal mucosal cells (Caco-Goblet cells), exposure of xyloglucan (plus gelatin and hibiscus and propolis extracts, Utipro^®^, a medical device for the management of UTIs) was non-cytotoxic and protected tight junctions (as TEER-*trans*-epithelial electrical resistance- increase in comparison with untreated cells) and preserved the paracellular flux between the apical and basolateral compartments (Lucifer yellow, LY, values similar to untreated cells) [16]. These results highlight the protective barrier properties of xyloglucan on the intestinal mucosa, which can help to maintain or restore intestinal barrier integrity in intestinal disorders.

**Figure 3:**
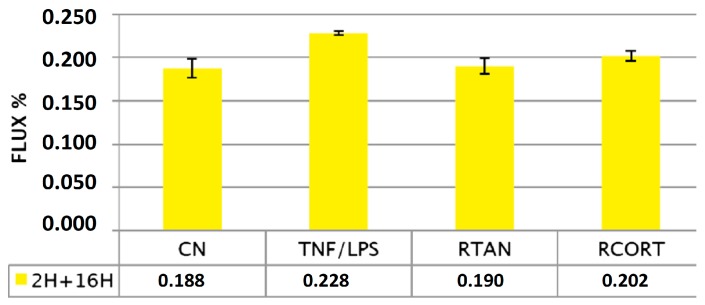
Preservation of paracellular flux by Rhinosectan^®^ between the apical and basolateral compartments of MucilAir. Preservation of paracellular flux reflects the integrity of the mucosal barrier. Lucifer yellow (LY) permeability after 2 h of pre-treatment with Rhinosectan^®^ (30 µL) and 16 h of exposure to pro-inflammatory compounds (LY flux (%) values). LY Flux = (RFUbl/RFUap) × 100, where RFUbl are fluorescent units detected at the basolateral compartment and RFUap are units detected at the apical part compartment. CN: negative control (pre-treatment with saline solution), without presence of pro-inflammatory compounds; TNF/LPS: pre-treatment with saline solution and exposure to pro-inflammatory compounds (TNF-α 500 ng/ml plus LPS from *Pseudomonas aeruginosa* 10, 0.2 mg/mL); RTAN: pre-treatment with Rhinosectan^®^ (30 µL) and 16 h of exposure to pro-inflammatory compounds; RCORT: pre-treatment with Rhinocort^®^ spray (budesonide) (30 µL) and 16 h of exposure to pro-inflammatory compounds [1].

Xyloglucan was also studied in the airway tissue model Mucil-Air-Nasal, as Rhinosectan^®^ spray, a medical device containing xyloglucan as its main ingredient, developed to restore the physiological functions of the nasal epithelial mucosa forming a film that protects the mucosa from different pathogens and allergens [16]. Results obtained showed that exposure of MucilAir with Rhinosectan^®^ protected cell tight junctions (increases in TEER of 13.1% vs. −6.3% with saline solution after 1 h of exposure), and preserved the paracellular flux, even after exposure with pro-inflammatory compounds (TNF-α and LPS from *Pseudomonas aeruginosa* 10). The exposure with the glucocorticoid budesonide (Rhinocort) produced higher LY fluxes than Rhinosectan^®^ (0.202%, *p* < 0.05) (Figure 3) [1]. Results of confocal immunofluorescence microscopy showed that after treatment with the pro-inflammatory mixture, Rhinosectan^®^ produced a slight relocation of zona occludens-1 in the cytosol compartment (while Rhinocort induced expression of zona-occludens-1), maintaining the localization of occludin (similarly to negative control) [1].

These results show the differences in the effect produced between xyloglucan (non-pharmacological) and the pharmacological agent budesonide, with barrier properties attributed to xyloglucan, preserving tight junctions and paracellular flux, in comparison with a stronger effect attributed to budesonide, producing an increased expression of tight junctions.

### 5.2. Anti-Adhesive Properties in Caco-Goblet Cells and Uroepithelial Cells

In Caco-Goblet cells, xyloglucan and gelatin decreased the bacterial adsorption of *E. coli*, particularly in the homogenate mucus compartment [16]. In this model, the exposure of xyloglucan also prevented the adhesion of two strains of uropathogenic *E. coli* (UPEC) (expressing type 1 fimbriae and expressing P fimbriae) [17]. These results support the use of xyloglucan, together with other components such as hibiscus and propolis, for the management of UTIs, since it is able to decrease the adhesiveness of the intestinal reservoir of UPEC, the first step to proliferation and migration from the intestinal tract to the perineal region and, therefore, to the urinary tract [70].

Moreover, xyloglucan and the other components of Utipro^®^ (hibiscus and propolis extracts) also prevented bacterial contact in an uroepithelial cell model (RWPE-1 cells), thus highlighting the utility of hibiscus in propolis in the management of UTIs, since they are systemically absorbed and can exert these protective effects directly in the urinary tract. Xyloglucan, in contrast, is not absorbed and its barrier effect is exerted in the intestinal tract, reducing the intestinal reservoirs of UPEC, in the case of UTIs management [17].

### 5.3. Anti-Invasive Properties in Caco-Goblet Cells

In the model of intestinal mucosa with Caco-Goblet cells, 4 h of preventive treatment with xyloglucan, together with hibiscus and propolis, also reduced invasion of *E. coli*, in comparison with monolayers only exposed with *E. coli*, thus highlighting the protective barrier properties against bacterial adhesion and subsequent invasion [16]. These results highlight that xyloglucan acts by forming a protective intestinal biofilm that is able to decrease the *E. coli* load in the intestinal lumen, thus preventing the development of UTIs by decreasing the number of UPEC in the stools and reducing the likelihood of contamination of the urethra-genital tract [25].

### 5.4. Wound Healing on Confluent Conjunctival Cells

In a culture of human conjunctival cells, xyloglucan influenced cell adhesion to laminin: at low laminin concentrations, xyloglucan increased cell adhesion, while at higher laminin concentrations xyloglucan inhibited cell adhesion to the glycoprotein. These results suggest that xyloglucan could facilitate cell adhesion by increasing the cell affinity for laminin, as occurs for other structures of the plasma membrane. This synergism, however, seems to be lost at higher concentrations. These findings suggest a concentration-dependency in which 1.0% might have a beneficial effect on conjunctival cell adhesion, suggesting that xyloglucan may be involved in corneal healing in vivo [22].

These results support the development of medical devices containing xyloglucan for the management of ophthalmological disorders.

### 5.5. Effects on Keratinocytes and Fibroblasts

In human skin keratinocytes and fibroblasts, xyloglucan improved keratinocyte and fibroblast proliferation, promoted the cell cycle and stimulated migration and intracellular enzyme activity, thus highlighting a possible role of xyloglucan in skin regeneration [71].

These results support the development of medical devices containing xyloglucan for the management of dermatological disorders.

## 6. Animal Studies with Xyloglucan and other Mucosal Protectors

### 6.1. Xyloglucan in a Model of Intestinal Injuries

In a study performed in Wistar rats, xyloglucan (12.5 mg/kg) was orally administered and 2 h later LPS from *E. coli* were injected via the intraperitoneal route. Jejuneal strips were collected 6 h later for in vitro tight junction permeability measurement. In another experiment, xyloglucan was administered orally associated or not with gelatin or co-administered with cholera toxin into isolated jejuna loops in anesthetized rats. Assessment of water secretion induced by cholera toxin was performed 2 h later [6,72].

Xyloglucan significantly decreased the LPS-induced increase in permeability by 81.8% (*p* < 0.01) and the subsequent increase in mucosal myelo-peroxidase activity by 63.2%. When orally administered 4 h earlier (12.5 mg/kg) or 12 h earlier with gelatin (250 mg/kg), xyloglucan suppressed the water secretion induced by cholera toxin. Co-administered locally with cholera toxin at doses of 0.75 and 1.25 mg/loop, xyloglucan decreased (67%) or suppressed, respectively, the secretory effects of cholera toxin [6,72].

These results demonstrate the protective barrier properties of xyloglucan on the intestinal mucosal cells, preserving them from bacterial virulence factors such as LPS or cholera toxin known to produce mucosal disruption. These favourable in vivo effects of xyloglucan correlate with the results of clinical trials obtained in adult and paediatric patients with gastroenteritis of infectious and other origins, in which intestinal inflammation is also present [23,24] (see Section 7 of this review).

### 6.2. Xyloglucan in a Model of Dry Eye

In a model of dry eye in rabbits, xyloglucan, in an artificial tear formulation, showed the best overall results in comparison with hydroxypropylmethylcellulose, sodium hyaluronate or sodium polyacrylate. In the Schirmer test, xyloglucan formulation produced high scores after the fourth day of treatment. In the slit-lamp examination of the fluorescein-treated corneas, at all observation times, xyloglucan formulation produced a significantly lower percentage (*p* < 0.01) of stained eyes in comparison with dry eyes or with the formulation without xyloglucan. Ferming tests showed that xyloglucan was able to crystallize forming type I fern-like structures. These results support the use of xyloglucan as a polymeric adjuvant for artificial tear formulations and ophthalmic vehicles. It might be hypothesized that xyloglucan, due to its good muco-adhesive and mucomimetic properties, might be absorbed at the cornea-tear interface, thus binding a thicker aqueous layer and favouring the stability of the tear film. This mechanism might explain the good protection against corneal desiccation, evidenced by the fluorescein test. Low, progressive adsorption of xyloglucan at the corneal surface could be hypothesized to explain the relatively longer time required by the formulation to attain high Schirmer test scores, indicative of a good degree of ocular hydration [73].

These results support the development of medical devices containing xyloglucan for the management of ophthalmological disorders.

### 6.3. Xyloglucan in a Model of Corneal Lesion in Rabbits

In a model of corneal damage in albino rabbits, treatment with a xyloglucan formulation (1.0%) slightly but significantly increased the wound healing rate, in comparison with a hyaluronate formulation. Xyloglucan exerted a positive influence on cell adhesion to laminin, up to a certain laminin concentration, in consonance with the results obtained in vitro in a culture of human conjunctival cells [22].

### 6.4. Reticulated Protein, Hibiscus and Propolis to Reduce Intestinal E. Coli Load

The impact of the medical device containing reticulated protein, hibiscus and propolis on intestinal *E. coli* strains was evaluated in Wistar rats (with normal microbiota or streptomycin-pretreated animals, as a model of *E. coli* colonization). The oral administration of the medical device significantly reduced faecal *E. coli* and *Enterococcus* spp. levels, without affecting other targeted *Enterobacteriaceae*. This antagonistic effect was also confirmed in streptomycin-pretreated rats highly colonized with human commensal *E. coli* strains with uropathogenic potential [74].

These results confirm the ability of mucosal protectors to specifically reduce the uropathogenic *E. coli* reservoirs present at intestinal level, as observed in the previously described in vitro studies with xyloglucan [16,17]. Since we assume that xyloglucan and reticulated protein could have a similar barrier effect, similar animal studies and clinical studies in patients with or at risk of UTIs should also be performed with medical devices containing xyloglucan (such as Utipro^®^).

## 7. Clinical Studies with Xyloglucan in Gastroenteritis

### 7.1. Gastroenteritis in Children

**Figure 4:**
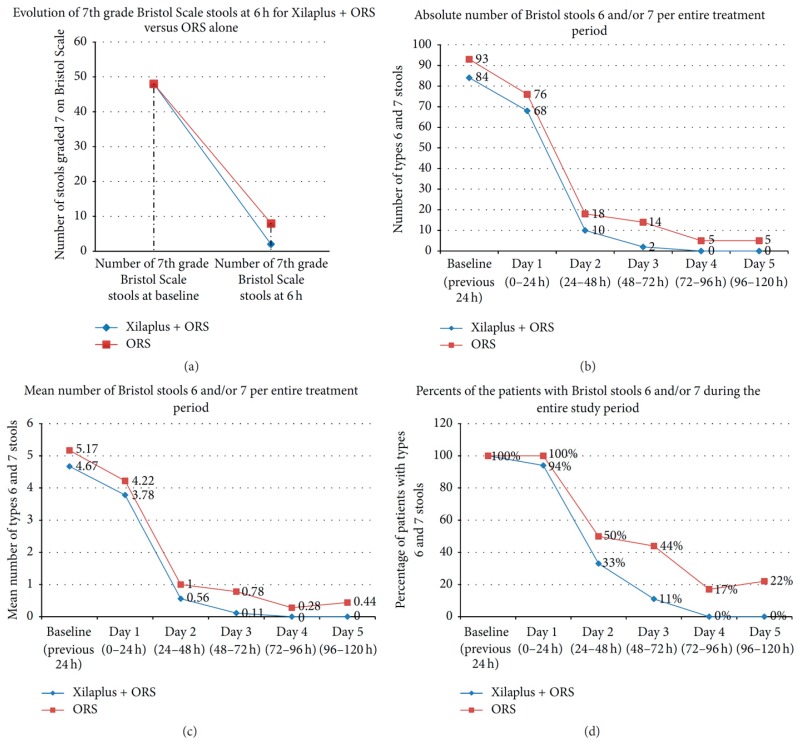
Evolution of type 6 and 7 Bristol Scale stools (the most dehydrating types of stools) in both groups of children (3 months–12 years) (Xyloglucan − Xilaplus- + ORS and ORS) over five days. (**a**) Evolution of the absolute number of type 7 stools during the first 6 h. (**b**) Evolution of absolute number of type 6 and 7 stools during the study period. (**c**) Evolution of mean number of type 6 and 7 stools during the study period. (**d**) Evolution of the percentage of patients with type 6 and 7 stools during the study period [24].

The effect at intestinal level of xyloglucan, in oral sachets (containing xyloglucan, gelatin, corn starch and magnesium stearate), plus oral rehydration solution (ORS), was assessed in a randomized, multicentre, ORS-controlled, open-label study in 36 children (*n* = 18 in each group) aged between 3 months and 12 years, diagnosed with acute gastroenteritis of infectious origin [24]. Patients receiving xyloglucan and ORS presented better symptom evolution than ORS-only treated patients, with a faster onset of action. At 6 h, xyloglucan produced a significantly higher decrease in the number of type 7 Bristol scale stools (0.11 vs. 0.44; *p* = 0.027). At Day 3 and 5, xyloglucan also produced a significant reduction in types 6 and 7 stools, in comparison with ORS alone (Figure 4). Xyloglucan plus ORS was safe and well tolerated [24].

These results confirm the favourable effects of xyloglucan in patients with gastroenteritis, particularly of infectious origin as in this case, although we consider that the absence of a double-blind design constitutes a limitation to clearly state the role of xyloglucan in paediatric gastroenteritis.

### 7.2. Gastroenteritis in Adults

A randomized, controlled, open-label, parallel group, multicentre clinical trial was performed to evaluate the effect of xyloglucan (oral capsules containing xyloglucan, gelatin, corn starch and magnesium stearate), in comparison with disomectite and *Saccharomyces* in 150 adult patients (*n* = 50 in each group) with acute diarrhoea due to different causes. Patients were randomized to receive a 3-day treatment. Symptoms (stools type, nausea, vomiting, abdominal pain and flatulence) were assessed by a self-administered ad-hoc questionnaire 1, 3, 6, 12, 24, 48 and 72 h after administration of the first dose [23].

**Figure 5:**
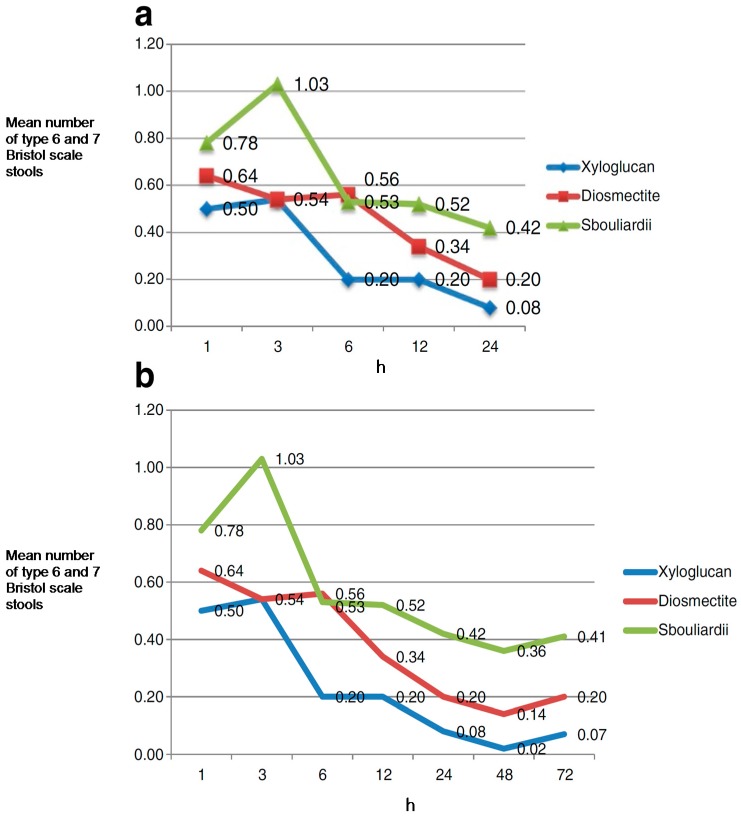
Clinical evolution of diarrhoeal symptoms (mean number of type 6 and 7 Bristol scale stools, the most dehydrating type of stools) among groups (xyloglucan, disomectite and *Saccharomyces boulardii*). (**a**) Mean number of type 6 and 7 stools during the first 24 h. (**b**) Mean number of type 6 and 7 stools during the study period (three-day treatment) [23].

During the first 24 h of treatment, xyloglucan presented a faster onset of action, with an improvement in diarrhoeal symptoms, measured as the mean number of Bristol scale type 6 and 7 stools, in comparison with the diosmectite and *S. bouliardii* groups. Accordingly, in the xyloglucan group the highest reduction in the number of type 6 and 7 stools was observed at 6 h, with an effect that was statistically significant compared with diosmectite group (*p* = 0.031). A greater improvement was observed in xyloglucan-treated patients in comparison with the *S. bouliardii* group at 12 and 24 h (Figure 5) [23]. Xyloglucan was the most efficient treatment in reducing the percentage of patients with nausea throughout the study period, particularly during the first h (from 26% at baseline to 4% after 6 and 12 h); and in reducing abdominal pain, with a sustained improvement throughout the study. As also observed in children, xyloglucan was well tolerated, without the occurrence of adverse events [23].

These results confirm the role of xyloglucan in the management of gastroenteritis of different origins, although we should note the need for double-blind studies.

## 8. Clinical Studies with Mucosal Protectors in Irritable Bowel Syndrome

The use of medical devices containing other film-forming agents, such as reticulated proteins, has also been assessed, in combination with a prebiotic mixture of vegetable oligo- and poly-saccharides, in patients with diarrhoea-predominant IBS [48].

In a randomized, placebo-controlled, double-blind, parallel group, multicentre, clinical trial, patients were randomly assigned to receive the combination of oligo- and poly-saccharides and reticulated protein and placebo (four oral tablets/day over 56 days) [48]. A total of 128 adult patients with diarrhoea-predominant IBS were randomized to receive either tablets containing the combination (*n* = 63) or placebo (*n* = 65). A significant improvement in symptoms during the study was observed in patients treated with the combination between visit 2 (at 28 days) and visit 3 (at 56 days) concerning abdominal pain (*p* = 0.0167) and flatulence (*p* = 0.0373). A statistically significant increase in the quality of life of patients treated with reticulated protein from baseline to visit 3 (*p* < 0.0001) was also detected. Treatment with this combination was safe and well tolerated [48].

Since in diarrhoea-predominant IBS, an altered intestinal barrier is a key feature, associated with immune activation and clinical symptoms [75], the use of mucosal protectors constitutes a new alternative for more efficient control of symptoms in these patients.

Based on these favourable results observed with reticulated proteins, we consider that it might also be worth assessing in a clinical trial the effect of xyloglucan in combination with a prebiotic mixture of vegetable oligo- and poly-saccharides in patients with IBS, particularly diarrhoea-predominant.

## 9. Clinical Studies with Mucosal Protectors in Urinary Tract Infections

### 9.1. Early Treatment of Patients with Symptoms of Urinary Tract Infections

In a double-blind, placebo-controlled trial in 60 adult patients (*n* = 30 in each group) with one or more symptoms of UTI (dysuria, urgency, suprapubic pain and/or urine organoleptic changes), the effect of a medical device containing a reticulated protein (gelatin), plus propolis and hibiscus administered orally over five days were evaluated. Results obtained showed that the risk ratio of patients who needed antibiotic treatment was significantly lower in the group treated with the medical device. During the study, only 10% of patients treated with the medical device required antibiotic treatment, versus 33.3% in the placebo group (*p* = 0.028). The medical device was also superior to the placebo in terms of clinical course, which was assessed based on the mean adjusted change scores for dysuria, urgency, suprapubic pain and organoleptic urine changes. An improvement in all the symptoms in the group treated with the medical device was observed after treatment completion. The treatment was safe and well tolerated [26].

### 9.2. Prevention of Uncomplicated Cystitis Recurrences

In a randomized, double-blind, placebo-controlled clinical trial in 78 adult women with acute cystitis symptoms, the effect of the oral administration of a medical device containing reticulated protein, hibiscus and propolis over six months were assessed. No recurrence was observed after the first month of follow-up in the group treated with the medical device. After 6 months, symptomatic recurrence among patients treated with the medical device was reduced by 19.4% in comparison with the placebo group (*p* = 0.015). The number of micturitions among patients treated with the medical device was significantly reduced, in comparison with the placebo group, at Day 3 and 20 [27].

Based on these results and on in vitro data obtained with xyloglucan [16,17], there is a need to assess xyloglucan, in combination with propolis and hibiscus, for the management of UTIs, for the prevention of recurrences and in patients with early symptoms of UTIs, in randomized, controlled clinical trials.

## 10. Clinical Studies with Xyloglucan in Nasal Disorders

### Xyloglucan-Based Nasal Spray in Rhinosinusitis

**Figure 6:**
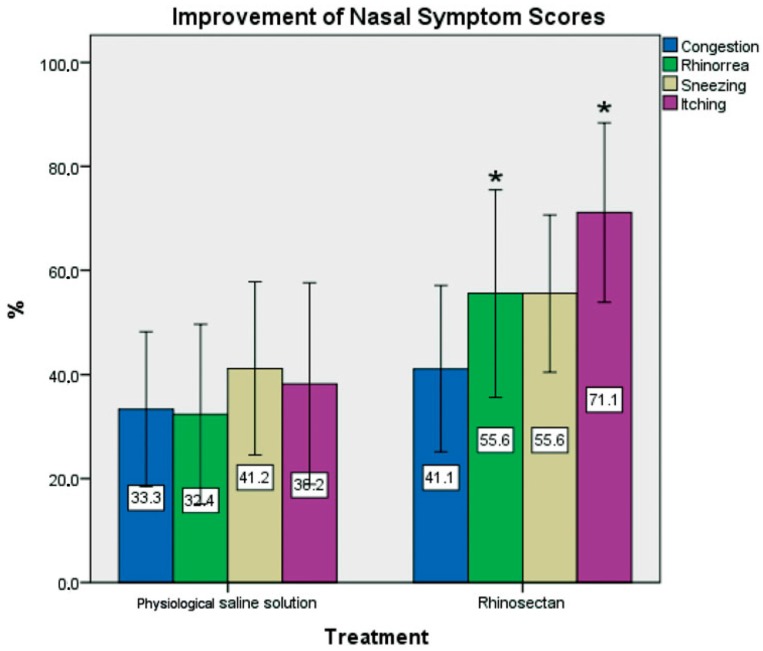
Percentage improvement in individual symptom scores from baseline to the end of treatment (2 weeks) in both groups of patients with rhinosinusitis (acute recurrent or chronic) (Rhinosectan^®^ and physiological saline spray). Administration consisted of two sprays per nostril four times daily. * *p* < 0.05, Rhinosectan^®^ vs. physiological saline spray at the end of treatment (day 15). TNSS (Total Nasal Symptom Score) was used to evaluate the severity of symptoms (congestion, rhinorrhoea, sneezing and itching) at baseline and at end of treatment, with a 4-point Likert scale [none, mild, moderate, severe] [15].

In a randomized, double-blind trial controlled with saline solution in 40 patients (*n* = 20) with itching, nasal congestion or continuous sneezing (Total Nasal Symptom Score—TNSS), the effect of a xyloglucan-based nasal spray (Rhinosectan^®^) in comparison with physiological saline spray for 2 weeks was assessed. A significant improvement in the rhinosinusitis severity index was observed only with xyloglucan (*p* < 0.05). At the end of treatment, mean scores were significantly lower in the xyloglucan group versus saline group for TNSS (3.60 vs. 5.40, *p* < 0.05) and rhinosinusitis severity index (7.55 vs. 6.45, *p* < 0.05), rhinorrhoea and itching (both *p* < 0.05). More patients in the xyloglucan than in the saline group reported the absence of nocturnal awakening from baseline to end of treatment. In the group treated with the Rhinosectan^®^, an improvement of more than 40% compared with baseline was observed for all four individual nasal symptoms, whereas only sneezing improved to this extent in the physiological saline group (Figure 6). No rescue medication was used. Both treatments were well tolerated [15].

These results are in consonance with those observed in an organotypic D airway tissue model (MucilAir), where the application of Rhinosectan^®^ created a protective physical barrier able to prevent the contact of nasal cells with allergens and triggering factors [1]. These studies confirm the role of xyloglucan in the form of Rhinosectan^®^ for the management of nasal respiratory diseases, such as rhinitis and rhinosinusitis, in line with current recommendations [3,69].

## 11. Clinical Studies with Xyloglucan in Ophthalmology

### Xyloglucan for the Treatment of Dry Eye Syndrome

In a randomized, open-label, comparative study in 30 adult patients with dry eye symptoms, the effect of a xyloglucan ocular formulation (0.5% and 1%) vs. a hyaluronic acid formulation (0.2%) during a period of 90 days was assessed. Xyloglucan showed greater benefits than hyaluronic acid in some of the subjective visual analogue scale (VAS) scores (blinking trouble, ocular burning and sensation of foreign bodies). These results suggest that xyloglucan at 0.5% and 1% offers at least equivalent relief to hyaluronic acid at 0.2% for dry eye syndrome. All treatments demonstrated optimal tolerability and were considered to be suitable for frequent use in the therapy of dry eye [28].

## 12. Clinical Studies with Xyloglucan in Infantile Colic

In a pilot study in 46 infants aged 3--16 weeks with infantile colic, the administration of xyloglucan plus heat-killed *Lactobacillus reuteri* SGL01 and *Bifidobacterium brevis* SGB01 significantly decreased the mean duration of crying episodes, in comparison with a lactase dietary supplement. These results suggest a role of xyloglucan in the management of infantile coli, although further research in larger studies is needed [76].

## 13. Concluding Remarks and Future Perspectives

Disruption of the epithelial barrier function is being associated with a variety of diseases, mainly at intestinal level, but also affecting the respiratory epithelium [2,3,5]. In this context, non-pharmacological approaches such as xyloglucan, with demonstrated protective barrier properties, are proposed as new alternatives for the management of a wide range of diseases, for which mucosal disruption and, particularly, tight junction alterations, is a common characteristic.

Xyloglucan, a natural biodegradable polysaccharide derived from the tamarind seed, possesses a “mucin-like” molecular structure that confers mucoadhesive properties. These mucoadhesive properties allow xyloglucan formulations to act as a barrier that is capable of reducing bacterial adherence and invasion and preserving tight junctions and paracellular flux, as observed in different in vitro cell models [1,16,17]. Xyloglucan has also been seen to have a role in corneal healing and in skin regeneration [22,71]. These favourable in vitro results have been correlated with favourable results in clinical trials in patients with gastroenteritis [23,24], nasal disorders [15] and dry eye syndrome [28]. Similar mucosal protectors containing reticulated proteins have also proved to be useful for the treatment of IBS [48] and UTIs [26,27]. Based on the similar film-forming protective effects between xyloglucan and other mucosal protectors, medical devices containing xyloglucan can also be expected to have a role in IBS and UTIs, although randomized clinical trials should be performed to demonstrate this.

Based on the results described in this review, we can conclude that medical devices containing compounds with protective barrier properties, such as xyloglucan, offer a non-pharmacological alternative for the management of different diseases characterized by mucosal epithelium disruption.

However, further research is required to assess the role of xyloglucan in other related processes, as the possible inhibition of bacterial biofilms and, in consonance, its possible favourable effects in disorders in which bacterial biofilms are a key characteristic, such as catheter-associated UTIs (CAUTIs). The possible role of xyloglucan in dermatological diseases with alterations of structural proteins and lipids of the stratum corneum and epidermal tight junctions leading to a diminished skin barrier function, such as atopic dermatitis [77], would deserve further research in in vitro and in vivo studies. Prevention and management of certain microbial infections, at intestinal and respiratory level, and also at vaginal level, with xyloglucan medical devices are also future research projects.

In conclusion, xyloglucan, endowed with film-forming protective barrier properties, is a safe non-pharmacological alternative for the management of different diseases such as gastrointestinal and nasal disorders.
